# Direct-to-Consumer Drug Advertisements Can Paradoxically Increase Intentions to Adopt Lifestyle Changes

**DOI:** 10.3389/fpsyg.2016.01533

**Published:** 2016-10-03

**Authors:** Maya B. Mathur, Michael Gould, Nayer Khazeni

**Affiliations:** ^1^Department of Biostatistics, Harvard UniversityBoston, MA, USA; ^2^Quantitative Sciences Unit, Department of Medicine, Stanford University, StanfordCA, USA; ^3^Department of Research and Evaluation, Kaiser Permanente Southern CaliforniaPasadena, CA, USA; ^4^Division of Pulmonary and Critical Care Medicine, Stanford University Medical CenterStanford, CA, USA; ^5^Center for Health Policy and Center for Primary Care and Outcomes Research, Stanford UniversityStanford, CA, USA

**Keywords:** direct-to-consumer advertising, risk compensation, health psychology, boomerang effect, attitude change

## Abstract

**Background:** Direct-to-consumer (DTC) prescription drug advertisements are thought to induce “boomerang effects,” meaning they reduce the perceived effectiveness of a potential alternative option: non-pharmaceutical treatment via lifestyle change. Past research has observed such effects using artificially created, text-only advertisements that may not adequate capture the complex, conflicting portrayal of lifestyle change in real television advertisements. In other risk domains, individual “problem status” often moderates boomerang effects, such that subjects who currently engage in the risky behavior exhibit the strongest boomerang effects.

**Objectives:** We aimed to assess whether priming with real DTC television advertisements elicited boomerang effects on perceptions of lifestyle change and whether these effects, if present, were moderated by individual problem status.

**Methods:** We assembled a sample of real, previously aired DTC television advertisements in order to naturalistically capture the portrayal of lifestyle change in real advertisements. We randomized 819 adults in the United States recruited via Amazon Mechanical Turk to view or not view an advertisement for a prescription drug. We further randomized subjects to judge either lifestyle change or drugs on three measures: general effectiveness, disease severity for a hypothetical patient, and personal intention to use the intervention if diagnosed with the target health condition.

**Results:** Advertisement exposure induced a statistically significant, but weak, boomerang effect on general effectiveness (*p* = 0.01, partial *R*^2^ = 0.007) and did not affect disease severity score (*p* = 0.32, partial *R*^2^ = 0.0009). Advertisement exposure elicited a reverse boomerang effect of similar effect size on personal intentions, such that advertisement-exposed subjects reported comparatively higher intentions to use lifestyle change relative to drugs (*p* = 0.006, partial *R*^2^ = 0.008). Individual problem status did not significantly moderate these effects.

**Conclusion:** In contrast to previous literature finding large boomerang effects using artificial advertisement stimuli, real television advertisements elicited only a weak boomerang effect on perceived effectiveness and elicited an unexpected reverse boomerang effect on personal intentions to use lifestyle change versus drugs. These findings may reflect real advertisements’ induction of descriptive norms and self-efficacy; future research could address such possibilities by systematically manipulating advertisement content.

## Introduction

Direct-to-consumer (DTC) advertising of prescription drugs is a flourishing industry. Pharmaceutical companies spent an estimated $4.5 billion on DTC promotion in the United States in 2009 (Mackey et al., 2015). However, there is longstanding debate over whether US federal law should more stringently regulate DTC advertising or even prohibit it entirely, as is currently the case in all countries except the US, New Zealand, and Brazil. In particular, the issue of how these advertising campaigns could shift lay consumer perceptions and health behavior has attracted a flurry of controversy (Wilkes et al., 2000; Hollon, 2005; Adams and Gables, 2016): DTC drug advertisements might beneficially raise health awareness among lay consumers (Dubois, 2003) or, alternatively, might encourage overtreatment, overprescription, and inappropriate use (Hollon, 2005).

Epidemiological research on the effect of DTC advertising on behavioral and economic outcomes has yielded mixed results. Patients were more likely to request a DTC-advertised drug in Sacramento, CA, where DTC advertising is legal, than in Vancouver, Canada, where DTC advertising is illegal (Mintzes et al., 2003). Additionally, patients with higher self-reported exposure to advertising requested more advertised drugs (Mintzes et al., 2003). The most heavily DTC-advertised drugs in 1999 saw the highest 1-year growth in sales (Findlay, 2001). However, monthly expenditures on DTC advertising are positively associated with physician diagnoses and prescriptions only for certain drugs and pharmaceutical classes, with other drugs and classes showing no association or a negative association (Calfee et al., 2002; Zachry et al., 2002). Specifically regarding lifestyle choices, DTC television advertising for statin drugs from 2001 to 2009 was associated cross-sectionally with increases in visits to fast-food restaurants, but also with increased exercise frequency (Niederdeppe et al., 2016).

From a cognitive perspective, increases in the frequency of DTC advertising on television in the US from 2001 to 2007 were cross-sectionally associated with increases in guilt surrounding failure to engage in healthy lifestyle behaviors (Kruger et al., 2015), which could usefully catalyze behavior change or reflect a detrimental reduction in self-efficacy, an important component of successful behavior change (Ajzen, 1991). Interpretation of these mixed findings is further complicated by the usual caveats of observational research and the fact that advertising expenditure clearly is not determined in isolation, but rather is responsively adjusted to market conditions and sales figures (Dulisse, 1997). Internal marketing research on DTC advertising conducted by pharmaceutical companies is not publicly available.

Given that the primary objective of DTC advertising is to increase pharmaceutical sales, a point of particular concern is how these advertisements could affect lay perceptions of non-pharmaceutical lifestyle change to treat and prevent disease. Exposure to messages promoting risk-reducing remedies can decrease risk perceptions and increase risky behavior (termed the “boomerang effect” or “risk compensation”). Such effects have been demonstrated in randomized experiments in diverse risk domains, including smoking, irresponsible credit card usage, fat consumption, and behaviors promoting online identity theft (Bolton et al., 2006). Paradoxically, individuals who currently engage in target risky behaviors (those with high “problem status”) are often most prone to boomerang effects. A study using an artificially created, text-only advertisement for a cholesterol-lowering drug demonstrated boomerang effects on perceptions of the effectiveness of lifestyle change, and the effect was moderated by individual problem status (Bolton et al., 2008).

The portrayal of lifestyle change in actual DTC television advertising is complex. A content analysis of 38 television advertisements found that a high proportion (18.4%) portrayed lifestyle changes as insufficient for controlling the target health condition, and not a single advertisement described lifestyle as an alternative to drugs (Frosch et al., 2007). On the other hand, 53% of advertisements portrayed the protagonist engaging in physical activity, which might promote a positive view of lifestyle change by fostering descriptive norms for a high prevalence of healthy lifestyle behaviors. Descriptive norms are an individual’s perception of the extent to which others engage in a target behavior, and they are an important predictor of both health-promoting and health-detrimental behaviors (Webb and Sheeran, 2006). An experimentally constructed social network demonstrated striking causal effects of peers’ health-related behaviors on subsequent individual behavior change (Centola, 2010). Quasi-experimental and observational evidence using naturalistic social networks similarly showed that individual development of obesity (Christakis and Fowler, 2007) and use of alcohol (Kremer and Levy, 2008) are associated with the individual’s proximity to peers who demonstrate these behaviors. Together, these findings on descriptive norms and peer effects suggest mechanisms by which portrayals of healthy lifestyles in DTC advertising could, in theory, improve perceptions of lifestyle change.

However, DTC advertising does not paint a uniformly positive picture of lifestyle change: a census study of television advertisements for cholesterol-lowering drugs found that many (73%) made explicit claims about the efficacy or inefficacy of diet or exercise, with most of these cases (65%) sending mixed messages via statements such as, “Diet and exercise are important, but when they aren’t enough…” The frequent and conflicting portrayal of lifestyle change in actual DTC television advertising stands in contrast to portrayals in the simple advertisement stimuli previously shown to demonstrate boomerang effects. For example, these advertisement stimuli did not in any way mention lifestyle change. Additionally, randomized past research has typically used printed advertisements; the comparatively richer multimedia content and length of television advertisements suggests they might contain more complex portrayals of lifestyle change than their printed counterparts. Thus, assessing the existence of boomerang effects in a sample of real DTC television advertisements is an important step toward understanding their public health implications.

In light of the limitations of previous research, we performed a high-powered randomized experiment to directly assess possible boomerang effects of DTC advertising on judgments of lifestyle change and drugs. We experimentally manipulated exposure to one of six real DTC television advertisements for three health conditions (high cholesterol, diabetes, and depression) and examined the effect of advertisement exposure on laypeople’s estimates of effectiveness and personal intentions to use lifestyle change versus drugs to alleviate the target health condition. Because our aim was to assess the effect of exposure to real advertisements rather than to specific elements of content, we assembled a sample of advertisement stimuli representing a variety of health conditions and major drug brands rather than selecting advertisements for predetermined characteristics or for particular references to lifestyle. To maximize external validity, stimuli were actual, previously aired advertisements.

## Materials and Methods

### Design Overview

**FIGURE 1:**
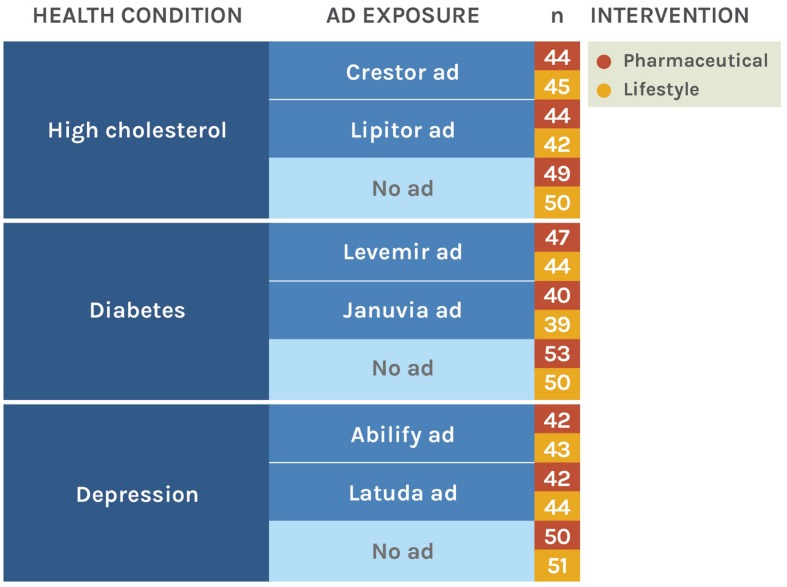
**Experimental conditions and sample sizes**. Values in the final column represent the number of subjects in that condition in the final, analyzed sample.

This study was approved by the Stanford University IRB, and the requirement for written consent was waived. We randomized subjects using a three-factor (health condition X advertisement exposure X judgment of lifestyle change versus drugs) between-subjects design (**Figure 1**). Randomization was conducted internally via an online questionnaire design software (2013, Qualtrics, Provo, UT, USA) and was balanced across the 18 experimental conditions.

At the beginning of the questionnaire, subjects either viewed a drug advertisement for the target health condition (**Table 1**) and completed an attention-check question, or in the no-advertisement conditions, proceeded directly to the effectiveness questions. (Because of the inherent difficulty of identifying a truly “neutral” counterpart to the advertisement stimuli, particularly with regard to norm induction, we did not present any video clip to subjects in no-advertisement conditions.) Advertisements were viewed once. Then, each subject judged the effectiveness of either lifestyle change or prescription drugs for the target health condition. The between-subjects manipulation of intervention type eliminated the possibility that subjects would experience order effects or directly compare the two types of interventions. We tested all questionnaire materials for comprehension on pilot samples and, in the experimental sample, probed for confusion or technical problems.

**Table 1:** Content of Advertisement Stimuli

Brand, year aired, generic name	Plot	Explicit references to lifestyle	Duration (min:sec)
**High cholesterol**			
Crestor (2013) *Rosuvastatin calcium*	Man eagerly watching television sees an ad for Crestor and celebrates. His room is full of sports-like memorabilia bearing Crestor’s name and logo colors.	None	1:00
Lipitor (2012) *Atorvastatin calcium*	Cyclist compares “steep risks” he took as teenager to risk of not taking Lipitor for high cholesterol. Vignettes of protagonist tossing football with son, riding bicycle, going to amusement park with family.	Protagonist states, “Why kid myself? Diet and exercise weren’t lowering my cholesterol enough. Now I’m eating healthier, exercising more, taking Lipitor.”	1:00
**Diabetes**			
Levemir FlexPen (2013) *Insulin detemir*	Woman describes the changes she must make after having been diagnosed with diabetes. Doctor hands her advertised product. Woman drives with family to attend grandmother’s birthday party, poses for family photo.	Protagonist states, “There’s a lot I have to do: check my blood sugar, eat better, start insulin.”	1:15
Januvia (2008) *Sitagliptin*	Vignettes of woman repeatedly climbing stairs, couple preparing vegetables, and woman walking through the park.	The message “Today I chose to take the stairs” appears onscreen.	0:53
**Depression**			
Abilify (2014) *Aripiprazole*	Animated woman “feels stuck” and “still struggles” with depression despite taking an antidepressant, a tablet of which follows her throughout the ad. She consults her doctor, who recommends adding Abilify. Protagonist is shown smiling at a work meeting and interacting with family.	None	1:31
Latuda (2014) *Lurasidone HCl*	Vignettes of woman brushing her hair, riding bike, speaking to doctor, watching children at playground, going to work, having lunch with friend, walking dog, and walking on beach with family.	None	1:31

### Setting and Participants

We sampled 819 subjects via Amazon Mechanical Turk, a crowdsourcing platform allowing workers to complete brief online tasks in exchange for pay. By contractual agreement with Mechanical Turk, workers must be at least 18 years of age. Workers tend to be somewhat younger, more educated, and lower-income than the US general population, but are demographically more representative than typical university-based research samples (Paolacci et al., 2010). Studies performed on Mechanical Turk yield high-quality data, minimize experimental biases, and successfully replicate the results of behavioral studies performed on traditional samples (Paolacci et al., 2010). Given the nearly ubiquitous reach of DTC advertising in the US, its public health implications may extend far beyond patients with the targeted health conditions; we therefore did not restrict the sample to individuals with specific health conditions. The task title and description were vague to minimize sampling bias and demand characteristics. We sampled US workers with excellent performance history (>95% of previous online tasks “approved” as high-quality by requester) and compensated each subject $0.25.

### Advertisement Stimuli

We selected three target health conditions (high cholesterol, diabetes, and depression) that are widespread, familiar to laypeople, and at least somewhat responsive to lifestyle change, specifically of dietary or exercise habits (Gillies et al., 2007; Thomas and Elliott, 2009; Hooper et al., 2012; Rimer et al., 2012). For each of these three health conditions, we obtained approximately 1-min advertisements for two major brands, for a total of six advertisements (**Table 1**). We obtained them from an online pharmaceutical advertising database^1^, manufacturer websites, and YouTube.com. All advertisements had aired since 2008 on United States television networks.

### Attention Check

For subjects who viewed an advertisement, we included the multiple-choice attention check question “According to the video, what is the purpose of the drug?”, to which only one of seven possible answers was correct. We eliminated from analysis all subjects responding incorrectly to this question (an *a priori* decision).

### Outcome Measures

We collected both direct and indirect outcome measures, comprising two measures of perceived effectiveness and one measure of personal intention to use either lifestyle change or drugs (Weinstein, 1998).

To measure perceived **general effectiveness**, we asked, “In your opinion, how effective is/are [*intervention*] at helping with [*health condition*]?”, where words in brackets depended on the subject’s experimental group. Lifestyle change was described as “healthy lifestyle changes (such as improving nutrition, controlling weight, and increasing exercise),” corresponding to recommendations published by the Centers for Disease Control and Prevention [CDC] (Singer, 2009; Centers for Disease Control and Prevention, 2013). Drugs were described as “[*health condition*] drugs.” Subjects responded on an integer-valued, 100-point visual analog scale (VAS) with four evenly-spaced verbal labels: “Very Ineffective,” “Ineffective,” “Effective,” and “Very Effective” on which higher values indicated higher effectiveness.

For the second outcome measure, **disease severity score**, subjects read instructions explaining that the lower the score, the better. They were instructed to “Imagine a patient with [*health condition*] (score 161) who begins using [*intervention*]. Please estimate the patient’s [*health condition*] score after 6 months of using [*intervention*].” Subjects responded on a 180-point VAS.

For the **intention** outcome, we asked, “Imagine you were recently diagnosed with [*health condition*]. How likely would you be to use [*intervention*]?” Subjects responded on a VAS identical to that used for the general effectiveness measure.

### Problem Status

We operationalized individual problem status via two questions gaging current adherence to exercise and diet recommendations: “Do you currently engage in at least: 75 min per week of vigorous-intensity physical exercise, OR 150 min of moderate-intensity exercise?” and “On a typical day, do you eat a healthy diet (rich in vegetables, limited in refined sugar, limited in fatty and fried foods, low in highly processed junk food)?” Subjects currently meeting criteria for both exercise and a healthy diet were classified as “low problem status,” while subjects failing to meet either criterion were classified as “high problem status.”

### Chronic Television Exposure

Previous observational research has documented an association between chronic exposure to advertising and prescription drug requests (Mintzes et al., 2003). As a proxy, we measured chronic television exposure with the question: “On a typical day, how many hours of television do you watch?”

### Demographic and Behavioral Measures

We collected data on the following demographic and behavioral characteristics: age, sex, body mass index (BMI) (calculated from height and weight), diet, exercise, education level, number of prescription drugs taken regularly, and duration of television watched on a typical day. These factual questions appeared at the end of the survey to prevent priming and stereotype threat effects on the outcome measures (Steele and Aronson, 1995; Burgess et al., 2010).

### Statistical Analysis

We performed all analyses in R (Version 3.0.2, multiple contributors, Vienna, Austria) (Fox and Weisberg, 2010; R Core Development Team, 2014) and defined statistical significance at an alpha level of 0.05. All tests were two-sided.

### Primary Analyses

To investigate baseline perceptions of perceived effectiveness and intention, we first restricted analysis to subjects who did not view an advertisement. We used *t*-tests to assess differences in each outcome measure between subjects judging lifestyle change and those judging drugs.

To assess the effect of advertisement exposure, we fit a linear regression model with normally distributed error terms to predict each outcome measure (Supplementary Methods, Model 1). Each model included main effects for advertisement exposure (subject viewed, versus did not view, an advertisement), type of health intervention judged (subject judged lifestyle change versus drugs), health condition (subject judged the outcome measure for high cholesterol, diabetes, or depression). We were primarily interested in the interaction of advertisement exposure with type of health intervention judged, as it estimates a possible differential effect of advertisement exposure for lifestyle change versus drugs. For the general effectiveness and personal intention measures, a negative interaction would indicate that advertisement exposure disproportionately improved perceptions of drugs versus lifestyle change. For the disease severity measure, lower scores indicate greater effectiveness, so a positive interaction would indicate the same. We also included interactions accounting for the possibility that some health conditions are perceived as especially conducive to either lifestyle change or drugs.

### Secondary Analyses

To assess whether individual problem status moderates a potential boomerang effect of advertisement exposure, we also analyzed a secondary model including a three-way interaction term of problem status.

If perceived ineffectiveness is a significant barrier to adopting a healthy lifestyle, then individuals who do not currently follow lifestyle recommendations for exercise and diet would be expected to perceive lifestyle as less effective than individuals who do follow recommendations. We investigated this possibility by restricting analysis to only subjects who did not view an advertisement. Among this subset, we fit a linear regression model (Supplementary Methods, Model 2) predicting each outcome measure with type of health intervention judged and current self-reported lifestyle (the subject meets both exercise and dietary requirements or does not). The interaction between the two variables is the term of primary interest, as it represents a differential effect of intervention type between subjects with high and low problem status.

## Results

### Sample Characteristics

**FIGURE 2:**
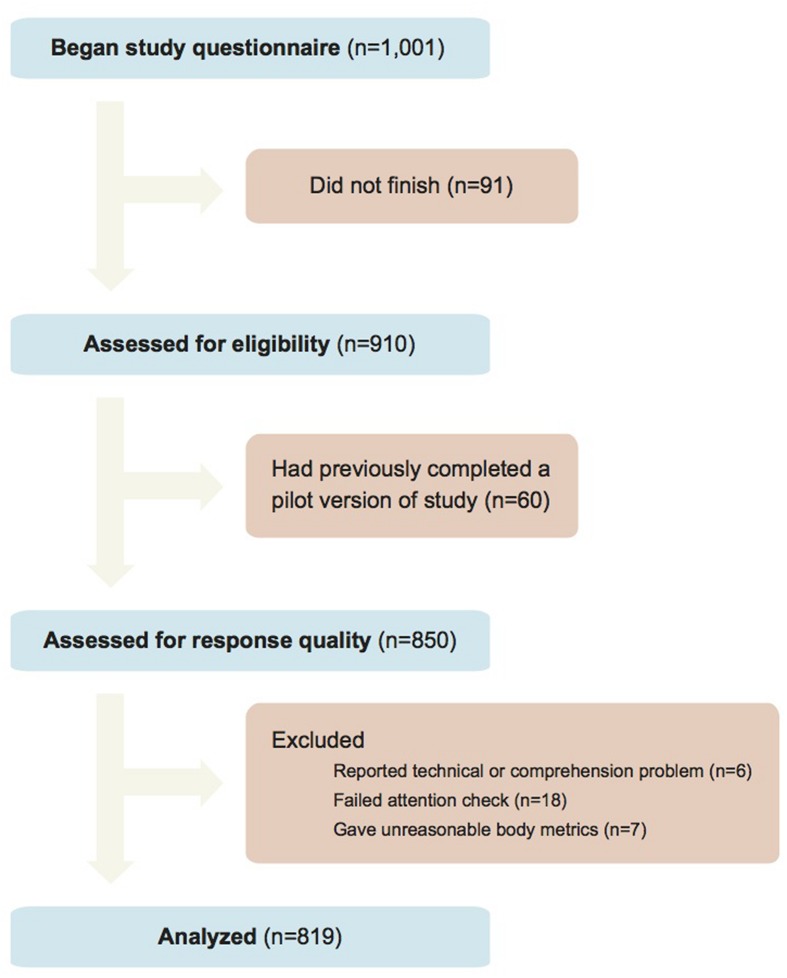
**Study flow diagram**.

Data quality was excellent. Reported comprehension of all questionnaire items was very high, with only four subjects reporting any confusion. Of 850 subjects who finished the study and who had not previously completed a pilot trial, we excluded 31 responses (3.6%) due to quality problems (6 reported technical or comprehension problems, 18 failed the attention check question, and 7 gave unreasonable body metrics; **Figure 2**). Sensitivity analyses that instead included all subjects yielded nearly identical results. Thus, the final analyzed sample size was 819; the breakdown by condition is presented in **Figure 1**. Negligible differences in sample size across the conditions are due to dropout.

Subjects were predominantly male (62.0%), young (median age 27 years), and relatively educated (e.g., 38.2% held a 4-year college degree). Slightly more than half met national standards for diet (55.8%) and for exercise (55.6%), and 24.1% reported regularly taking a prescription drug. Subjects were on average borderline overweight (median BMI 25.0 kg/m^2^) and reported watching a median of 2.0 h of television daily (Supplementary Table S1). Although we did not collect data on ethnic characteristics in this study, our recent work using nearly identical recruitment procedures (Mathur and Reichling, 2016) found the ethnic background of Mechanical Turk users to be approximately 80% Caucasian, 11% Hispanic, 6% Southeast Asian, 5% Native American, and <5% each: East Asian, South Asian, Pacific Islander, Black, and Middle Eastern (percentages sum to more than 100% due to multiracial subjects).

### Baseline Perceptions of Lifestyle Change and Drug

We assessed baseline perceptions of lifestyle and pharmaceutical intervention effectiveness among the *n* = 303 subjects who did not view a drug advertisement (**Table 2**), finding that subjects considered lifestyle change to be more effective, or as effective, as drugs for all outcome measures. Specifically, perceived general effectiveness was much higher for lifestyle than pharmaceutical interventions (mean VAS units 80.2 and 62.8, *p* < 0.001). A similar pattern occurred for personal intention (means 71.8 and 62.5, *p* = 0.004). However, disease severity score did not differ significantly between intervention types (means 106.4 and 104.0, *p* = 0.45).

**Table 2:** Perceived effectiveness of lifestyle change versus drugs by advertisement exposure.

	General effectiveness	*p*-value	Disease severity score	*p*-value	Intention to use	*p*-value
**Did not watch advertisement**						
Lifestyle (*n* = 151)	80.2 (15.1)		106.4 (27.6)		71.8 (22.3)	
Drug (*n* = 152)	62.8 (20.4)	<0.001	104.0 (28.4)	0.45	62.5 (32.9)	0.004
**Watched advertisement**						
Lifestyle (*n* = 257)	77.6 (17.2)		102.8 (29.0)		78.8 (20.8)	
Drug (*n* = 259)	66.3 (18.5)	<0.001	104.4 (27.6)	0.52	58.8 (32.4)	<0.001

*Data reported are means and SDs. *n* = 819*.

We also assessed whether baseline perceptions of lifestyle change and drugs depended on whether subjects themselves followed lifestyle recommendations for exercise and diet. Personal adherence to lifestyle recommendations was unrelated to relative perceived effectiveness of lifestyle change and drug: Among subjects not exposed to an advertisement, there was no significant interaction of meeting lifestyle criteria with intervention type for either the general effectiveness (*b* coefficient = 5.44, *p*-value = 0.20, partial *R*^2^ = 0.004) or the disease severity score measure (*b* = -6.50, *p* = 0.32, partial *R*^2^ = 0.003)^2^.

### Boomerang Effects of Advertisement Exposure

**FIGURE 3:**
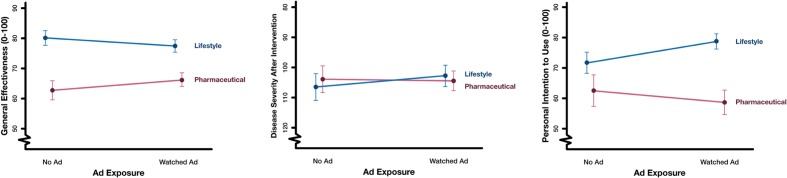
**Main effects and interaction of intervention type with advertisement exposure**. Values are presented in visual analog scale (VAS) units. The scales for general effectiveness and intention ranged from 0 to 100, with higher scores indicating higher effectiveness or intention. The scale for disease severity score ranged from 0 to 180, with a starting score of 161 before the intervention and lower scores indicating better health; the vertical axis in the disease severity plot is reversed for consistency. Error bars represent 95% confidence intervals. *n* = 819.

In primary regression analyses (**Table 3**; **Figure 3**), the endpoint of interest was the interaction coefficient of advertisement exposure with intervention type, which measures the relative impact of advertisement exposure on perceptions of lifestyle change versus drugs (the boomerang effect). We found that advertisement exposure increased perceived general effectiveness slightly more for drugs versus lifestyle change. However, the opposite was true for the personal intention measure: advertisement exposure disproportionately increased personal intention to use lifestyle versus drugs. That is, there was a small, but significant, negative interaction for the general effectiveness outcome (*b* = -6.2, *p* = 0.01, partial *R*^2^ = 0.007), no significant interaction for the disease severity score outcome (*b* = -3.9, *p* = 0.32, partial *R*^2^ = 0.0009), and a positive interaction for the personal intention outcome (*b* = 10.6, *p* = 0.006, partial *R*^2^ = 0.008). Omitting the main effect of health condition and interactions of health condition with intervention type, or stratifying on health condition (Supplementary Figure S1), did not appreciably change results. Including three-way interaction terms of health condition with intervention type suggested some significant differences in the interaction of interest across diseases, but these were not consistent across outcome measures.

**Table 3:** Boomerang effects of advertisement exposure on perceptions of lifestyle and drug effectiveness

Outcome	Variable	Coefficient (*SE*)	95% CI	*p*-value
**General effectiveness**	Intervention judged			
	Drugs	*Ref*	*Ref*	*Ref*
	Lifestyle	19.5 (2.6)	[14.4, 24.7]	<0.001
	Ad exposure			
	No ad	*Ref*	*Ref*	*Ref*
	Watched ad	3.6 (1.8)	[0.1, 7.0]	0.04
	Health condition			
	Cholesterol	*Ref*	*Ref*	*Ref*
	Diabetes	9.2 (2.1)	[5.1, 13.2]	<0.001
	Depression	-6.6 (2.1)	[-10.7, -2.5]	0.002
	**Lifestyle ^∗^ Watched ad**	**-6.2 (2.5)**	**[-11.1, -1.3]**	**0.01**
	Lifestyle ^∗^ Diabetes	-6.3 (2.9)	[-12.1, -0.6]	0.03
	Lifestyle ^∗^ Depression	0.6 (2.9)	[-5.1, 6.4]	0.83
**Disease severity score**	Intervention judged			
	Drugs	*Ref*	*Ref*	*Ref*
	Lifestyle	1.3 (4.1)	[-6.8, 9.5]	0.75
	Ad exposure			
	No ad	*Ref*	*Ref*	*Ref*
	Watched ad	0.2 (2.8)	[-5.24, 5.7]	0.94
	Health condition			
	Cholesterol	*Ref*	*Ref*	*Ref*
	Diabetes	-10.7 (3.3)	[-17.2, -4.3]	0.001
	Depression	-18.2 (3.3)	[-24.7, -11.7]	<0.001
	**Lifestyle ^∗^ Watched ad**	**-3.9 (4.0)**	**[-11.6, 3.9]**	**0.32**
	Lifestyle ^∗^ Diabetes	3.5 (4.7)	[-5.6, 12.7]	0.45
	Lifestyle ^∗^ Depression	-0.1 (4.7)	[-9.3, 9.0]	0.98
**Personal intention**	Intervention judged			
	Drugs	*Ref*	*Ref*	*Ref*
	Lifestyle	16.3 (4.0)	[8.5, 24.1]	<0.001
	Ad exposure			
	No ad	*Ref*	*Ref*	*Ref*
	Watched ad	-3.5 (2.7)	[-8.8, 1.8]	0.19
	Health condition			
	Cholesterol	*Ref*	*Ref*	*Ref*
	Diabetes	20.8 (3.2)	[14.6, 27.0]	<0.001
	Depression	-6.3 (3.2)	[-12.5, -0.04]	0.05
	**Lifestyle ^∗^ Watched ad**	**10.6 (3.8)**	**[3.1, 18.0]**	**0.006**
	Lifestyle ^∗^ Diabetes	-18.6 (4.5)	[-27.4, -9.8]	<0.001
	Lifestyle ^∗^ Depression	-1.3 (4.5)	[-10.1, 7.5]	0.77

*Coefficients are presented in visual analog scale (VAS) units. The scales for general effectiveness and intention ranged from 0 to 100, with higher scores indicating higher effectiveness or intention. The scale for disease severity score ranged from 0 to 180, with lower scores indicating lower disease severity. *n* = 819*.

### Individual Problem Status

To estimate the difference in potential “boomerang” effects between subjects with high versus low problem status, we fit three-way interactions of individual problem status (meeting both dietary and exercise requirements) with advertisement exposure and intervention type. We found no evidence for moderation by problem status: the interaction terms were non-significant for all outcomes (general effectiveness: *b* = -3.2, *p* = 0.54, partial *R*^2^ = 0.0004; disease severity: *b* = -0.4, *p* = 0.97, partial *R*^2^ < 0.0001; personal intention: *b* = -14.1, *p* = 0.07, partial *R*^2^ = 0.0006).

### Effect of Television Exposure

Because it appeared counter-intuitive that drug advertisements would not substantially increase perceived effectiveness or personal intention to use the advertised products, we performed *post hoc* secondary analyses assessing the role of time spent watching television. If subjects were already saturated by everyday exposure to DTC advertisements, then the lack of effect of a single experimental exposure to advertisements might belie a cumulative shift in perceptions as a result of chronic exposure to such advertisements.

We conducted two analyses to investigate this possibility. First, to assess whether subjects with little or no chronic exposure to DTC television advertisements would be sensitive to experimental exposure, we repeated analyses among only those subjects who reported watching no television on a typical day (*n* = 163). Advertisement exposure had no significant interaction with intervention for the general effectiveness (*b* = -3.2, *p* = 0.62, partial *R*^2^ = 0.008) or disease severity score measures (*b* = -13.2, *p* = 0.18, partial *R*^2^ = 0.007). As in the main analysis, there was a large *positive* interaction for the personal intention measure (*b* = 19.7, *p* = 0.02, partial *R*^2^ = 0.03).

Additionally, we investigated whether, among subjects who did not view an advertisement (*n* = 303), increased television-watching was associated with a more favorable perception of drugs versus lifestyle change. We fit a linear regression model (Supplementary Methods, Model 3) to predict each outcome measure with main effects and the interaction of the number of hours of television watched on a typical day with type of intervention judged (lifestyle change versus drugs).

A negative interaction between television-watching time and lifestyle change (versus drugs) would indicate that individuals watching more television had a relatively less favorable view of lifestyle (versus drugs) than individuals watching less television. In fact, there was no such interaction for any of the outcome measures (general effectiveness: *b* = -0.9, *p* = 0.34, partial *R*^2^ = 0.002; disease severity score: *b* = 0.2, *p* = 0.89, partial *R*^2^ < 0.0001; personal intention: *b* = -1.4, *p* = 0.33, partial *R*^2^ = 0.003). Therefore, regularly consuming more television, and by proxy more DTC television advertisements, did not appear to shift perceptions of relative effectiveness or personal intention to use lifestyle change versus drugs.

### Older Subjects

Because our sample was relatively young (median age 27 years), we performed a sensitivity analysis to assess whether the primary endpoint (the interaction of advertisement exposure with health intervention judged) differed by subject age by introducing a three-way interaction in analysis models. These coefficients were small and not statistically significant, suggesting that primary findings were fairly robust to differences in subject age. Power may have been limited by low variability in subject age.

## Discussion

To our knowledge, this is one of the first randomized experiments on the effects of real direct-to-consumer television advertisement exposure on viewers’ relative perceptions of lifestyle change and drugs to alleviate disease. Past experimental research has mostly used simple printed advertisements that may not have captured the complex, often conflicting portrayal of lifestyle change in real DTC television advertisements. Past observational research may have been limited by confounding, for example with sociological factors such as peer effects, whereas our design enables rigorous causal conclusions. The present study used a high-powered randomized design to estimate relative judgments of lifestyle change versus drugs and directly assess possible “boomerang effects” of DTC advertising exposure. In order to most closely capture the effect of real-life DTC advertising, stimuli were actual advertisements previously aired on US national television, and we did not manipulate advertisement content.

In contrast to previous findings, exposure to DTC advertisements induced only a weak boomerang effect on perceived general effectiveness and induced a reverse boomerang effect of similar magnitude on personal intentions. If subjects already viewed large numbers of similar advertisements in everyday life, a single experimental exposure might be insufficient to shift perceptions. However, we measured perceptions immediately after advertisement exposure, a method sensitive to even short-lived effects. Also, advertisement exposure had little effect among even subjects who watched no television. In contrast to previous observations that patients with higher self-reported exposure to advertising were more likely to request prescription drugs (Mintzes et al., 2003), we found that regular television consumption was not associated with perceptions of drug effectiveness, either in absolute terms or relative to lifestyle. These effects were not moderated by individual problem status. The discrepancy with previous observational findings could arise if DTC advertisements influence prescription drug requests through mechanisms other than perceived effectiveness and intention – for example, by raising disease awareness or increasing visits to physicians.

Unexpectedly, we found that advertisement exposure induced a small “reverse boomerang effect” on personal intentions to use lifestyle change relative to drugs. We suggest two possible explanations. First, a key proposed mechanism of the boomerang effect is that people may tend to associate prescription drug use with poor health, and this association may in turn reduce the viewer’s perceived ability to engage in healthy lifestyle change via reduced self-efficacy (Bolton et al., 2008). However, as we noted in our introduction, real DTC television advertisements frequently portray characters engaging in physically active behaviors not consistent with poor health; such portrayals may suppress or reverse detrimental effects on self-efficacy. Second, we speculate that these portrayals may additionally invoke beneficial descriptive norms regarding the prevalence of physical activity, convincing the viewer that “everyone is doing it.” Indeed, norm induction is a potent method of behavior change in many domains (Rivis and Sheeran, 2003). Although our sample of advertisements was not large enough to assess whether specific aspects of lifestyle portrayal moderated individual advertisements’ effects, such analyses of content would be valuable future directions. Additionally, future research could begin investigating our proposed mechanisms by assessing mediation by self-efficacy and descriptive norms.

Our research has limitations. Our sample was not a national random sample and generalizability is unknown, although as previously discussed, Mechanical Turk samples tend to more closely resemble national demographic characteristics than do traditional university study samples. Health-related lifestyle behaviors, and hence potentially also perceptions of their effectiveness, are known to differ by race. Therefore, the baseline perceptions of our predominantly Caucasian sample may not generalize to more racially diverse populations (Dong et al., 2013). We used self-reported measures of perceived effectiveness and personal intention, which may be subject to demand characteristics and self-promotion biases. To minimize these biases, we used uninformative study descriptions for recruiting and worded the questionnaire using neutral terms (Lavrakas, 2008). Future research could incorporate behavioral and implicit attitude measures to supplement the self-report measures used in our study (Greenwald et al., 1998). Additionally, perceptions of intervention effectiveness for a given health condition may be different among disease-affected versus healthy individuals. For example, the target health conditions may be more familiar or personally salient to older, at-risk individuals; however, sensitivity analyses suggested that findings did not differ by age. Because we were interested in considering boomerang effects in the context of public health implications of DTC advertising, we did not restrict sampling to subjects aﬄicted with the diseases of interest. However, future research could specifically recruit from these populations.

Framing our work within the broader national debate on the regulation of DTC advertising, our findings provide a useful perspective on a specific hypothesized negative effect of DTC advertisements on viewers. Several other factors should also be considered in federal decision-making regarding regulation of DTC advertisements, and indeed regarding whether these advertisements should be permitted at all. For example, the potential economic effects of DTC advertising on prescription drug prices require nuanced assessment. Pharmaceutical companies’ advertising expenses may trickle down to the consumer (Gagnon and Lexchin, 2008), potentially contributing to extremely high costs of medications in the United States (Tefferi et al., 2015); on the other hand, some speculate that advertising actually promotes beneficial market competition between pharmaceutical companies (Wilkes et al., 2000). Policymakers should ultimately use empirical evidence to weigh such economic and health-systems impacts as well as their cognitive impacts on the consumer, including those evaluated in our study.

## Conclusion

We find that DTC television advertisements can paradoxically induce a weak boomerang effect on perceived effectiveness while also inducing an unexpected “reverse boomerang effect” with a comparable effect size on personal intentions. Given the contrast between our findings and those of past experiments using artificially created print advertisements, we have outlined possible mechanisms by which the observed reverse boomerang effect might occur, informed by established theories of descriptive norm induction and self-efficacy. Future work could therefore use a large sample of real advertisements to investigate whether content features predict the extent to which a particular advertisement does or does not induce boomerang effects. By elucidating the paradoxical psychological effects of DTC advertising, such research could ultimately inform evidence-based policy on DTC advertising regulation.

## Research Transparency

All raw data, R code, and questionnaire materials (including files that can be directly imported into Qualtrics) are publicly available at https://osf.io/zxdjk/.

## Author Contributions

MM and NK conceptualized the study. MM collected data, planned and performed statistical analyses, and drafted the manuscript. NK and MG provided critical revisions to the manuscript.

## Conflict of Interest Statement

The authors declare that the research was conducted in the absence of any commercial or financial relationships that could be construed as a potential conflict of interest.

## Footnotes

1 http://AdPharm.net

2 In this case, the interaction for the personal intention measure would not be meaningful as it is in the other analyses. Interpretation of this question depends on whether one currently adheres to lifestyle recommendations: subjects who do not personally adhere to lifestyle recommendations would interpret the question as gauging their willingness to change their current lifestyle and those who do personally adhere to lifestyle recommendations would likely interpret the question as gauging their willingness to maintain their current lifestyle. (The inherent dependence of the question interpretation on the subject’s personal adherence to lifestyle is inconsequential in the other analyses due to randomization.)
