# Supplementary material for: Direct-to-Consumer Drug Advertisements Can Paradoxically Increase Intentions to Adopt Lifestyle Changes
Source: Front Psychol. 2016 Oct 3;7:1533. doi: 10.3389/fpsyg.2016.01533 (PMC5045930; doi:10.3389/fpsyg.2016.01533)
Supplement: Supplementary file 1 [file Data_Sheet_1.pdf]

Supplementary Material: Direct-to-Consumer Drug Advertisements Can Paradoxically Increase Intentions to Adopt Lifestyle Changes

Maya B. Mathur*, Michael Gould, and Nayer Khazeni
*Correspondence:
Maya B. Mathur:
mmathur@stanford.edu

All data and materials are available online at: https://osf.io/zxdjk/

1 SUPPLEMENTARY METHODS: REGRESSION MODELS

Model 1: Boomerang Effects (Primary Analysis)

To assess the effect of advertisement exposure, we fit the following linear regression for each outcome measure Y among all subjects:

$$\widehat{Y} = \widehat{\beta}_0 + \underbrace{\widehat{\beta}_1 X_{\text{lifestyle}}}_{\text{lifestyle vs. drugs main effect}} + \underbrace{\widehat{\beta}_2 X_{\text{ad exp.}}}_{\text{ad exp. main effect}} + \underbrace{\widehat{\beta}_3 X_{\text{lifestyle}} X_{\text{ad exp.}}}_{\text{differential effect of ad exposure on lifestyle vs. drugs}}$$

$$+ \underbrace{\widehat{\beta}_4 X_{\text{depress.}} + \widehat{\beta}_5 X_{\text{diab}}}_{\text{disease-specific main effects}} + \underbrace{\widehat{\beta}_6 X_{\text{lifestyle}} X_{\text{depress.}} + \widehat{\beta}_7 X_{\text{lifestyle}} X_{\text{diab}}}_{\text{disease-specific interactions}}$$

where $\widehat{Y}$ represents the predicted outcome measure, $X_{\text{lifestyle}}$ indicates judging lifestyle change (vs. drugs), $X_{\text{ad exp.}}$ indicates exposure to an advertisement (versus no advertisement exposure), $X_{\text{depress.}}$ indicates judging intervention effectiveness for depression (vs. high cholesterol), and $X_{\text{diab.}}$ indicates diabetes (vs. high cholesterol).

The interaction coefficient $\widehat{\beta}_3$ is the primary endpoint of interest, as it measures whether the effect of ad exposure is different for lifestyle change versus medication. Finally, the interaction coefficients $\widehat{\beta}_6$ and $\widehat{\beta}_7$ account for the possibility that some diseases are perceived as especially conducive to either lifestyle or pharmaceutical interventions.

Model 2: Association of Baseline Perceptions with Individual Problem Status (Secondary Analyses)

If perceived ineffectiveness is a significant barrier to adopting a healthy lifestyle, then individuals who do not currently follow lifestyle recommendations for exercise and diet would be expected to perceive lifestyle as less effective than individuals who do follow recommendations. We investigated this possibility

by fitting the following regression among the subjects who did not view an advertisement:

$$\widehat{Y} = \widehat{\alpha}_0 + \underbrace{\widehat{\alpha}_1 \mathrm{X}_{\text{lifestyle}}}_{\text{lifestyle vs. drugs main effect}} + \underbrace{\widehat{\alpha}_2 \mathrm{X}_{\text{healthy}}}_{\text{current problem status main effect}} +$$

$$\underbrace{\widehat{\alpha}_3 \mathrm{X}_{\text{lifestyle}} \mathrm{X}_{\text{healthy}}}_{\text{differential perceptions of lifestyle vs. drugs by problem status}}$$

$$+ \underbrace{\widehat{\alpha}_4 \mathrm{X}_{\text{depress.}} + \widehat{\alpha}_5 \mathrm{X}_{\text{diab}}}_{\text{disease-specific main effects}} + \underbrace{\widehat{\alpha}_6 \mathrm{X}_{\text{lifestyle}} \mathrm{X}_{\text{depress.}} + \widehat{\alpha}_7 \mathrm{X}_{\text{lifestyle}} \mathrm{X}_{\text{diab}}}_{\text{disease-specific interactions}}$$

where $\mathrm{X}_{\text{healthy}}$ indicates whether the subject's current lifestyle meets both dietary and exercise recommendations (low problem status). The interaction is the term of primary interest, as it represents a differential effect of intervention type between subjects with low vs. high problem status.

Model 3: Chronic Television Exposure (Secondary Analysis)

We fit the following regression for each outcome measure among subjects who did not view an advertisement:

$$\widehat{Y} = \widehat{\gamma}_0 + \underbrace{\widehat{\gamma}_1 \mathrm{X}_{\text{TV}}}_{\text{television time main effect}} + \underbrace{\widehat{\gamma}_2 \mathrm{X}_{\text{lifestyle}}}_{\text{lifestyle vs. drugs main effect}} +$$

$$\underbrace{\widehat{\gamma}_3 \mathrm{X}_{\text{lifestyle}} \mathrm{X}_{\text{TV}}}_{\text{differential perceptions of lifestyle vs. drugs by television-watching time}}$$

where X_{TV} represents the number of hours of television watched on a typical day.

2 SUPPLEMENTARY TABLES AND FIGURES

Demographic and Behavioral Characteristics

Variable	Full sample *(n=819)*	No-advertisement sample *(n=303)*	Advertisement-exposed sample *(n=516)*
**Age** (years)	27.0 [23.0 – 33.0]	27.0 [23.0 – 33.0]	27.0 [23.0 – 33.0]
**Sex**			
Male	62.0%	64.4%	60.7%
Female	38.0%	35.6%	39.3%
**BMI** (kg/m^2)	25.0 [22.1 – 29.4]	24.7 [22.3 – 29.2]	25.1 [21.9 – 29.4]
**Diet**[a]			
Healthy	55.8%	57.0%	57.0%
Unhealthy	44.2%	43.0%	43.0%
**Exercise**[b]			
Sufficient	55.6%	54.1%	56.4%
Insufficient	44.4%	45.9%	43.6%
**Education level**			
Less than high school	1.2%	1.3%	1.2%
High school	37.4%	36.3%	38.0%
2-year college	13.4%	12.2%	14.1%
4-year college	38.2%	39.3%	37.6%
Post-graduate degree	9.8%	10.9%	9.1%
**Takes prescription drug regularly**			
Yes	24.1%	27.4%	22.2%
No	75.9%	72.6%	77.8%
**Television watched on a typical day (hours)**	2.0 [1.0 – 3.0]	2.0 [1.0 – 3.0]	2.0 [1.0 – 3.0]

Table S1. For continuous variables, due to skewness, data reported are medians [first quartile – third quartile]. *a:* Sufficient exercise was defined as at least 75 minutes per week of vigorous-intensity physical exercise or 150 minutes of moderate-intensity exercise. *b:* Healthy diet was defined as rich in vegetables, limited in refined sugar, limited in fatty and fried foods, limited in highly processed junk food.

Main Effects and Interaction of Intervention Type with Advertisement Exposure, Stratified by Health Condition

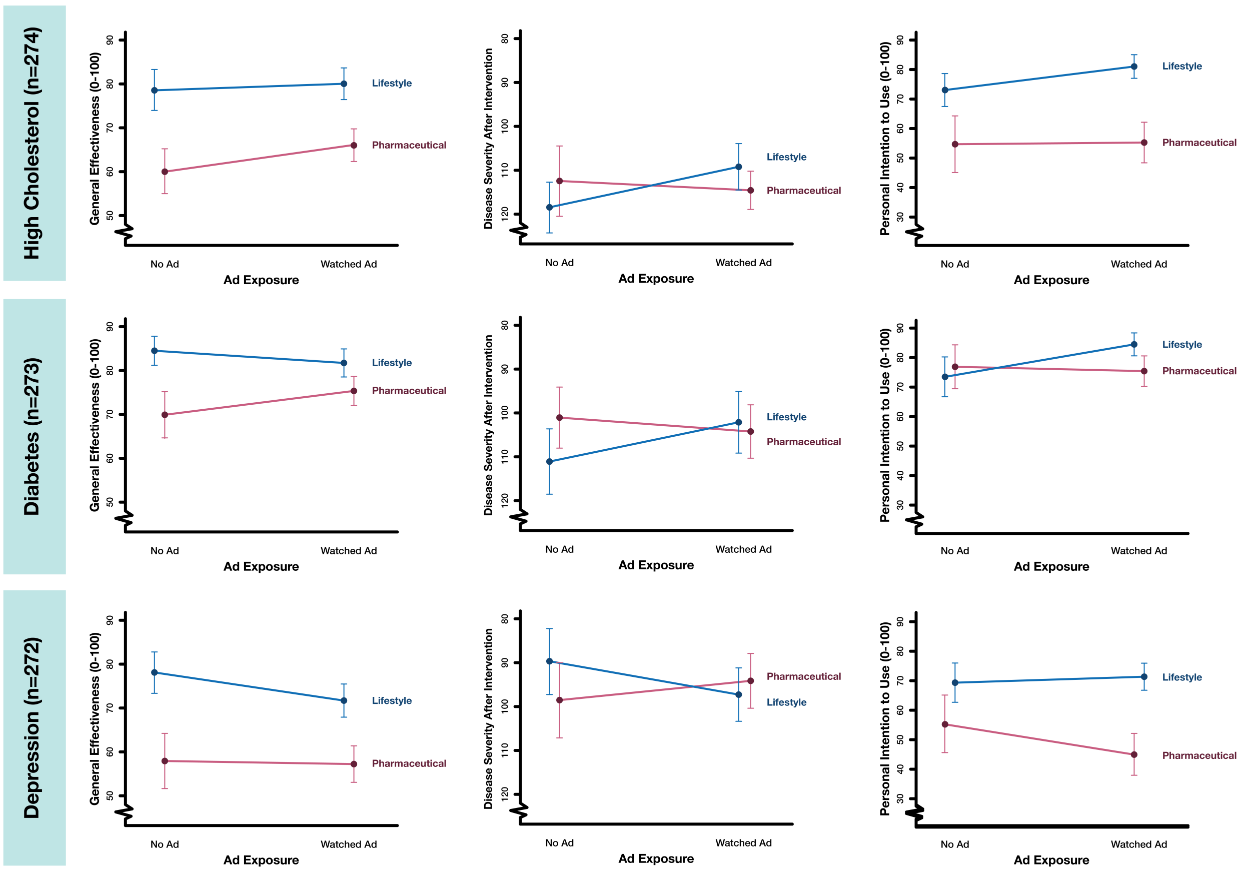

Figure S1. Values are presented in visual analog scale (VAS) units. The scales for general effectiveness and intention ranged from 0 to 100, with higher scores indicating higher effectiveness or intention. The scale for disease severity score ranged from 0 to 180, with lower scores indicating better health; the vertical axes in the disease severity plots are reversed for consistency. Error bars represent 95%confidence intervals.
